# The Interaction Between Two Worlds: MicroRNAs and Toll-Like Receptors

**DOI:** 10.3389/fimmu.2019.01053

**Published:** 2019-05-14

**Authors:** Recep Bayraktar, Maria Teresa Sabrina Bertilaccio, George A. Calin

**Affiliations:** ^1^Department of Experimental Therapeutics, The University of Texas MD Anderson Cancer Center, Houston, TX, United States; ^2^Center for RNA Interference and Non-Coding RNAs, The University of Texas MD Anderson Cancer Center, Houston, TX, United States; ^3^Department of Leukemia, The University of Texas MD Anderson Cancer Center, Houston, TX, United States

**Keywords:** microRNAs, Toll-like receptors, inflammation, TLR, TLR ligands

## Abstract

MicroRNAs (miRNAs) are critical mediators of posttranscriptional regulation via their targeting of the imperfect antisense complementary regions of coding and non-coding transcripts. Recently, researchers have shown that miRNAs play roles in many aspects of regulation of immune cell function by targeting of inflammation-associated genes, including Toll-like receptors (TLRs). Besides this indirect regulatory role of miRNAs, they can also act as physiological ligands of specific TLRs and initiate the signaling cascade of immune response. In this review, we summarize the potential roles of miRNAs in regulation of TLR gene expression and TLR signaling, with a focus on the ability of miRNAs bind to TLRs.

## Introduction

An efficient immune system is required for all multicellular organisms to detect and respond to pathogenic microorganisms or cells and/or tissue damage (1, 2). After discovery of the Toll-like receptor (TLR) family of pattern recognition receptors in the late 1990s, investigators showed that they recognize specific and distinct conserved endogenous and exogenous molecular patterns (3, 4). TLRs are crucial in recognition of microbial products, release of inflammatory mediators through the induction of transcription factors in immune response and inflammation and control of adaptive immune responses (5, 6). Emerging evidence has demonstrated that non-coding RNAs such as microRNAs (miRNAs) are involved in almost all known cellular processes, including innate, and adaptive immune responses, via modulation of gene expression (7–11). MiRNAs are secreted by several cell types, including tumor cells and macrophages within extracellular vesicles, such as exosomes and microvesicles; act as cell-to-cell communication vectors; and are taken up by recipient cells (12–17). Moreover, several miRNAs can bind to TLRs and initiate immune response by inducing immune and inflammatory gene expression. This review focuses on the inflammation-related miRNAs in the let-7 family, miR-21, miR-146b, and miR-155 and their involvement in TLR signaling pathways via regulation of TLRs and/or TLR signaling expression and binding to TLRs.

## TLRs and TLR Signaling

TLRs are evolutionarily conserved molecules that initiate the signaling cascade of immune response against a wide variety of pathogens (18). Moreover, TLRs are type I integral membrane proteins consisting of 10–30 leucine-rich repeats in the N-terminal portion of TLRs that participate in ligand recognition and a cytoplasmic domain of the Toll/interleukin (IL)-1 receptor (TIR) in the C-terminal portion of TLRs that is responsible for activation of downstream signaling. Both pathogen-associated and damage-associated molecular patterns can be recognized by different TLRs and subsequently trigger signaling transduction pathways through adaptor molecules (19, 20). Damage-associated molecular patterns are endogenous molecules released from stressed or dying cells. Depending on the type of cells and tissues damaged, they can be classified as protein damage-associated molecular patterns, such as heat shock proteins, high-mobility group box 1 protein, or non-protein damage-associated molecular patterns, such RNA and DNA (3, 21, 22). Pathogen associated-molecular patterns are exogenous molecules derived from pathogens such as bacteria, fungi, parasites, and viruses and can be recognized by TLRs, leading to activation of the TLR signaling cascade, which regulates the expression of inflammation-related genes such as IL-1 receptor-associated kinase (*IRAK1*), tumor necrosis factor (*TNF*) receptor-associated factor 6 (*TRAF6*), and type I interferon (*IFN*) (3, 21, 22). Various TLRs are primarily or selectively expressed in specific cell types, including immune cells such as lymphocytes, dendritic cells (DCs), macrophages, and neutrophils and non-immune cells such as epithelial cells and fibroblasts (23–26). Recent studies identified that TLRs are also expressed in tumor cells and their microenvironments that composed cancer-associated fibroblasts, tumor-associated macrophages, marrow-derived suppressive cells, and regulatory T cells, adipocytes, and immune cells (23, 27). In mammals, the TLR protein family currently comprises 13 members (humans, TLR1-10; mice, TLR1-9, and TLR11-13), with the TLRs in humans and mice having some functional differences (28–31). Based on the subcellular localization of TLRs, they can be broadly divided into two subgroups. Those in the first group, including TLR1, TLR2, TLR4, TLR5, TLR6, and TLR10, are expressed on the surface of cells and recognize microbially derived ligands. TLRs in the second group, including TLR3, TLR7, TLR8, and TLR9, are expressed intracellularly in vesicles such as endosomes and lysosomes and recognize microbial nucleic acids (32, 33). In contrast, TLR3 can be localized both on cell surfaces and in intracellular vesicles (34).

Upon activation of TLR signaling transduction pathways, TLRs interact with several TIR-containing intracellular adaptor molecules, including myeloid differentiation primary response gene 88 (*MYD88*), sterile alpha and TIR motif-containing protein 1, TIR domain-containing adaptor protein, TIR domain-containing adapter-inducing IFN-β (TRIF), TIR domain-containing adapter molecule 1 (TICAM1), and TICAM2, leading to transcription factor activation and ultimately causing the release of various proinflammatory cytokines, chemokines, and IFNs and activation of the adaptive immune system (35, 36). Depending on the adaptor protein recruited, TLR signaling can be activated via the MyD88-dependent pathway that leads to release of proinflammatory cytokines and a TRIF-dependent (MyD88-independent) pathway associated with production of IFN-β (37–40). TLR1, TLR2, TLR5, TLR6, TLR7, TLR8, and TLR9 signaling are activated by the MyD88-dependent pathway, which typically leads to activation of nuclear factor (NF)-κB, whereas TLR3 signaling is activated by the TRIF-dependent pathway. In contrast, TLR4 is activated by both pathways simultaneously (37–40).

## The Effects of miRNAs on TLR Expression and Signaling

**Figure 1:**
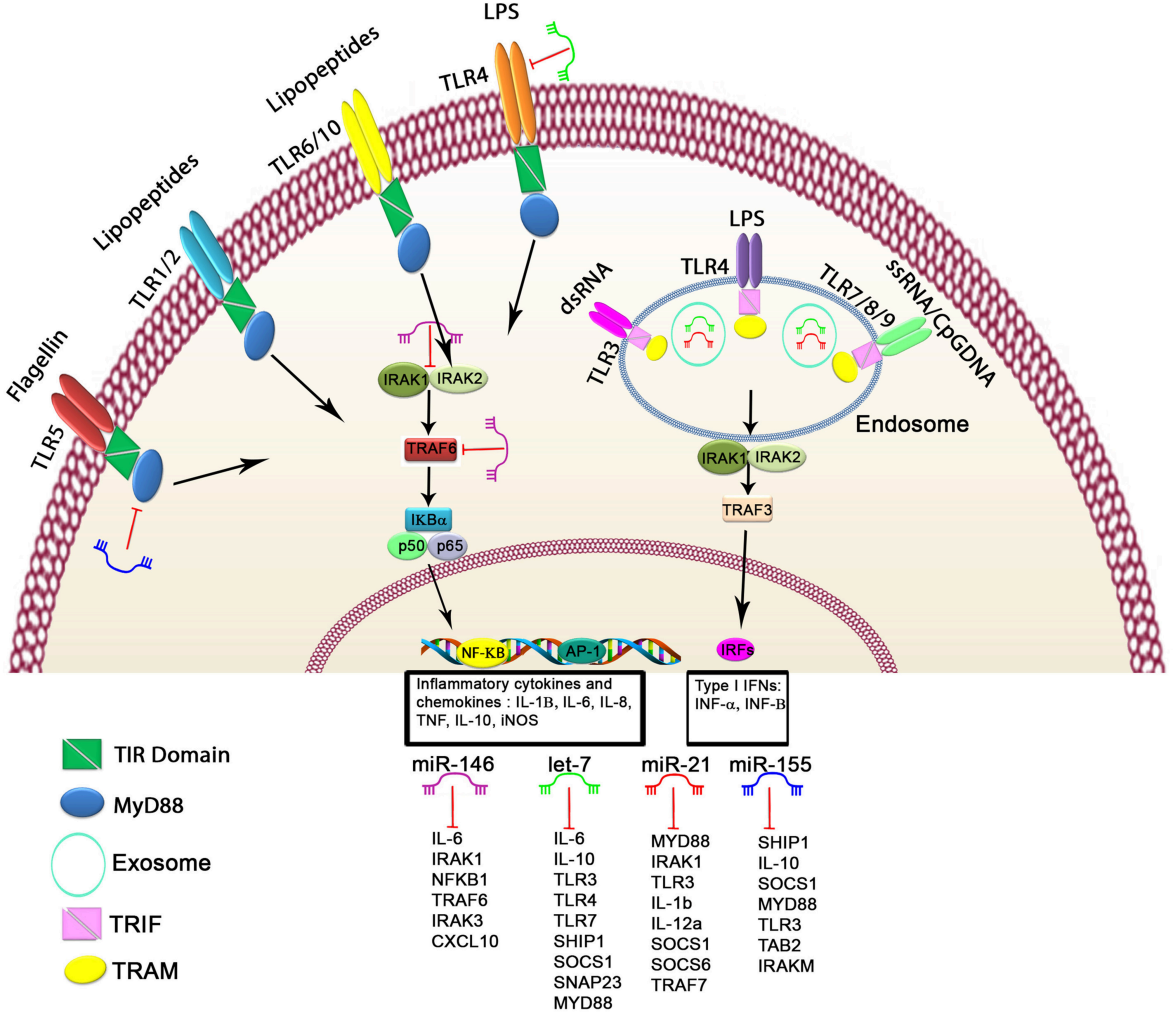
Schematic of the regulatory mechanism of miRNAs in TLR signaling. Cell surface and cytoplasmic TLRs can be regulated by several miRNAs, including let-7 family members, miR-21, miR-146, and miR-155. First, miRNAs can bind directly to 3′untranslated region of TLRs or TLR-related genes, leading to modulated expression of TLRs through posttranscriptional regulation of TLR signaling. Second, miRNAs serve as physiological ligands of TLRs, such as miR-21, let-7 family members, and miR-29a, which can activate TLR signaling and stimulate the release of inflammatory cytokines and IFN genes in some cell types. Functional studies have demonstrated that these miRNAs may participate in activation of TLR signaling through regulating the *NF-*κ*B* pathway and the production of inflammatory cytokines, which are shown here.

A growing number of reports have stated that specific epigenetic processes such as histone modifications, DNA methylation, and non-coding RNAs may regulate the transcriptional responses of TLRs (41–43). MiRNAs make up one of the well-characterized non-coding RNA families that generally bind to the 3' untranslated regions of their target messenger RNAs (mRNAs) to suppress translation or degradation of the mRNAs (44, 45). MiRNAs have a fundamental role in many biological processes, including apoptotic cell death, cell-cycle, tumorigenesis, and inflammation. Also, dysregulation of miRNAs has been associated with prognosis for and progression of multiple human diseases, including cancer (46–51). An increasing number of studies have demonstrated that several miRNAs, including miR-21, miR-146, miR-155, and let-7 family, target TLRs or proteins in TLR signaling pathways (Figure 1) that are involved in the regulation of various processes, such as inflammation, T-cell activation, cellular infiltration, and immunity development (52, 53). We have selectively listed recent miRNAs and their regulator roles on TLRs in Table 1.

**Table 1:** The regulatory effects of miRNAs on TLR signaling and the TLRs that regulate the miRNAs.

	**miRNA**	**Target/pathway**	**Cell or tissue type**	**Function**	**References**
The effects of miRNAs on TLR expression and signaling	let-7i	TLR4	Human cholangiocytes	Regulates TLR4 expression and contributes to immune responses against *C. parvum* infection	(54)
	let-7i	TLR4	Human monocytes	The let-7i mimic inhibits TLR4 expression	(55)
	let-7	TLR4	Human brain microvascular endothelial cells	Overexpression of let-7i reduces TLR4 expression and inflammation	(56)
	let-7/miR-98	SNAP23/TLR4	Human non-malignant biliary epithelial cells	The let-7 family reduces SNAP23 expression	(57)
	miR-155	TLR4 signaling	Murine Kupffer cells	Directly regulates expression of IRAK-M, SHIP1, SOCS1, and C/EBPβ	(58)
	miR-155	TLR3 signaling	Murine bone marrow macrophages	MiR-155 expression is dependent on TLR3/TRIF signaling	(59)
	miR-155	Caspase 3	Murine macrophages	MiR-155–mediated caspase 3 inhibition in LPS-activated macrophages suppresses apoptosis	(60)
	miR-155	TLR3	Avian macrophages	Inhibits IFN-β production in the TLR3 signaling pathway	(61)
	miR-155	CD1d	Human B cells	Directly targets CD1d upon TLR9 activation	(62)
	miR-155	TLR4 signaling	Murine ischemic cerebral tissue/microglial cells	Promotes TNF-α and IL-1β expression by upregulating TLR4 and downregulating SOCS1 and MyD88	(63)
	miR-155	MyD88 and SHIP1	Human primary monocyte-derived macrophages	Regulates downmodulation of MyD88 and SHIP1 expression and inhibits LPS-stimulated TNF-α secretion	(64)
	miR-155	SOCS1 and SHIP1	PBMCs	Suppresses expression of SOCS1 and SHIP1, which are negative regulators of TLR signaling	(65)
	miR-155	SHIP1	Murine macrophages	Represses SHIP1 expression and modulates ROS production	(66)
	miR-155	TGF-β and TLR3 signaling	Murine Kupffer cells and macrophages	Blocks the suppressive effect of IL-10 and TGF-β on TLR3 signaling	(67)
	miR-155	TNF-α and MCP1	Murine microglial cells	MiR-155 expression is induced by alcohol in the cerebellum in a TLR4-dependent manner	(68)
	miR-155	TLR3/4	Monocyte-derived macrophages	Restores infectivity in poly(I:C)-treated monocyte-derived macrophages	(69)
	miR-155	IRAK-M	Macrophages and PBMCs	Induces TLR7 stimulation and positively regulates IFN-α/β production in PDCs	(70)
	miR-155	SHIP1	Bone marrow-derived macrophages and PBMCs	IL-10 suppresses miR-155 expression in response to TLR4 stimulation	(71)
	miR-155	TAB2/TLR/IL-1	PBMCs	Controls the IL-1β pathway	(72)
	miR-21	PDCD4	Murine macrophages and human monocytes	Inhibits NF-κB activity and promotes IL-10 production	(73)
	miR-21	TLR4/ROS	Human primary lung cancer cells	Downregulation of miR-21 inhibits LPS-induced tumor growth	(74)
	miR-21	TLR4, IRAK3, and CXCL10	Human monocytes	Overexpression of miR-21 represses downstream transactivation of IL-1β and TNF-α	(75)
TLR signaling may modulate miRNA expression	let-7	CIS/TLR4	Human cholangiocytes	Activation of TLR4/MyD88 signaling downregulates miR-98 and let-7	(76)
	let-7	IL-6 and IL-10	Murine macrophages and human epithelial cells	Repression of let-7 activity relieves the cytokines IL-6 and IL-10	(77)
	let-7	TLR4	Murine neuroblastoma cells and macrophages	TLR4 regulates let-7 repression through KSRP	(78)
	miR-155	TLR4 signaling	Murine Kupffer cells	TLR4 signaling regulates miR-155 expression	(79)
	miR-155	SOCS1	Murine macrophages	Mediates TREM-1–induced effects on TNF-α, IL-1β, and IL-6	(80)
	miR-155	IL-10	Murine bone marrow-derived macrophages	Downmodulation of Ets2 expression leads to suppression of miR-155 expression by IL-10	(81)
	miR-155	TLR2/MyD88	PBMCs	MiR-155 expression is regulated by HMGB1 in a MyD88-dependent manner	(82)
	miR-155	TLR4	Bone marrow-derived macrophages	Tenascin-C drives LPS-induced miR-155 expression	(83)
	miR-155	SHIP1	PBMCs and bone marrow-derived macrophages	*F. tularensis* infection induces miR-155 expression in a TLR-dependent manner through downregulation of SHIP1	(84)
	miR-155	SOCS1	Murine macrophages	Progesterone-based treatment inhibits LPS-induced IL-6 production by decreasing the activity of miR-155	(85)
	miR-21	PTEN	PDCs	MiR-21–deficient PDCs produce low levels of IFN-α and IFN-γ	(86)
	miR-146	IRAK1 and TRAF6	Human acute monocytic leukemia cells	LPS induces NF-κB expression through a MyD88-dependent pathway, resulting in upregulation of miR-146	(87)
	miR-146	TLR4 signaling	Human umbilical vein endothelial cells	Ang-1 triggers upregulation of miR-146b	(88)
The ability of miRNAs to bind to TLRs	miR-21	TLR7 signaling	Macrophages/microglial cells	In extracellular vesicles, miR-21 can activate TLR7 signaling	(89)
	let-7b	TLR7	Murine neurons	Activates TLR7 and causes neurodegeneration	(15)
	miR-21	TLR7/8 signaling	HEK-293 cells and murine macrophages	Functions as a human TLR8 or murine TLR7 ligand	(13)
	miR-21		Hematopoietic cell lines and PBMCs	Functions as an endogenous agonist for TLR8	(90)
	miR-29a	TLR7/8 signaling	HEK-293 cells and murine macrophages	Functions as a TLR7/8 ligand	(13)
	let-7	TLR7	Murine macrophages and microglia	Functions as a ligand for murine TLR7	(15)

*CIS, cytokine-inducible Src homology 2-containing protein; KSRP, KH-type splicing regulatory protein; TREM-1, triggering receptor expressed on myeloid cells 1; ROS, reactive oxygen species; HMGB1, high-mobility group box 1; TGF, transforming growth factor; PDCs, plasmacytoid DCs*.

In one of the first studies demonstrating that miRNAs regulate immune response, researchers found that let-7i binds directly to TLR4 and regulates its expression in human cholangiocytes (54). In that study, infection of cultured cholangiocytes with *Cryptosporidium parvum* and lipopolysaccharide (LPS) stimulation of the cholangiocytes led to decreased let-7 expression via a MyD88/NF-κB–dependent mechanism, and low expression of let-7 was associated with upregulation of TLR4 in cholangiocytes. In concordance with this, upon *C. parvum* infection in non-malignant human biliary epithelial cells, inhibits expression of let-7 family miRNAs, including let-7i, let-7d, let-7f, let-7e, and miR-98, whereas induces the protein content of total SNAP23 and enhances phosphorylation of SNAP23. Activation of TLR4 signaling may induce SNAP23 protein expression by modulation of let-7-mediated gene regulation (57). Subsequently, investigators showed that let-7 and miR-98 target the 3' untranslated region of the cytokine-inducible Src homology 2-containing protein, resulting in translational repression of this protein in cholangiocytes, and that this may be associated with modulation of inflammatory responses in epithelial cells during microbial infection (76). In addition to this regulatory role of let-7 regarding TLR4 activation the inflammation-associated transcription factors NF-κB p50 and C/EBPβ can interact with the let-7 promoter region and repress transcription following microbial stimulus in human cholangiocytes (91).

MiR-155 has a well-characterized oncogenic role in tumorigenesis (92, 93), and aberrant expression and function of miR-155 have been associated with inflammation and affect immune cell functions at various levels by targeting inflammation-related genes, including TLRs (87, 94). This miRNA suppresses the expression of the adaptor protein TAB2 in the TLR/IL-1 signaling cascade, thereby regulating the feedback mechanism of IL-1β and other inflammatory cytokines produced during LPS-mediated DC activation (72). MiR-155 can also target suppressor of cytokine signaling 1 (SOCS1) and consequently modulate transcriptional expression of SOCS1 in LPS-activated Akt1^−/−^ murine macrophages (95). In addition, miR-155 and miR-M4 (virally encoded functional orthologs of miR-155) may target coding sequences of the TLR3 gene and regulate TLR3 expression in macrophages (61). In line with this, inhibition of miR-155 by antagomirs markedly increased TLR3 expression, whereas ectopic overexpression of miR-155 decreased IFN-β production in primary chicken embryo fibroblast cells (61). In another study, researchers found not only that miR-155 regulates TLR expression but also that miR-155 and caspase 3 mRNA can interact with AGO2 in LPS-activated murine macrophages (60).

MiR-21 is one of the multifunctional miRNAs and is mainly characterized by overexpression in many inflamed states, including lung inflammation in LPS-treated mice, allergic airway inflammation, and osteoarthritis (73, 96–98). Moreover, researchers detected high miR-21 expression in extracellular vesicles during simian immunodeficiency virus pathogenesis and increased miR-21 expression in mouse hippocampal neurons associated with neurotoxicity due to neuronal TLR7 expression (89). Furthermore, investigators showed that miR-21 expression was induced in murine macrophages by treatment with LPS, whereas proinflammatory protein PDCD4 expression was downregulated in these cells due to induction of miR-21 expression via the adaptor proteins MyD88 and NF-κB (73). In another study, miR-21 expression decreased in patients with primary graft dysfunction after lung transplantation, and incubation of human monocytes with bronchoalveolar lavage fluid obtained from patients with primary graft dysfunction induced miR-21 expression, suggesting that dysregulation of miR-21expression is a novel regulator of TLR signaling during development of lung injury (75). Another study demonstrated that activation of TLR4 by treatment with LPS induced miR-21expression in primary human lung cancer cells and reactive oxygen species production by these cells (74). A more recent study demonstrated that miR-21 was upregulated in plasmacytoid DCs and that miR-21 deficiency significantly impaired production of IFN-γ and IFN-α in response to *HSV-1* infection through targeting of the phosphoinositide 3-kinase/Akt/mammalian target of rapamycin signaling pathway in miR-21–knockout mice (86).

Importantly, an expression signature analysis of 200 miRNAs demonstrated that miR-146a/b, miR-132, and miR-155 were highly expressed in the acute monocytic leukemia cell line THP-1 after treatment with LPS as well as other microbial components and proinflammatory mediators (87). This finding suggests that miR-146 directly targets IRAK1 and TRAF6, which are key adapter molecules in the TLR4/NF-κB pathway (87). Furthermore, researchers found that miR-146 was significantly upregulated in hepatic stellate cells in mice infected with *Schistosoma japonicum* (99), is a negative regulator of NF-κB signaling in hepatic stellate cells, and acts by targeting TRAF6. Moreover, ectopic overexpression of the miR-146b-5p mimic significantly attenuated LPS-induced inflammatory responses and IRAK1 and TRAF6 expression in human umbilical vein endothelial cells (88).

Furthermore, miR-195 can regulate TLR2 expression through an indirect mechanism, as TLR2 expression was significantly reduced in miR-195–transfected THP-1 macrophages polarized toward the M1 phenotype (100). Treatment with LPS, synthetic lipid A, IL-2, IL-15, IL-1β, IFN-γ, and TNF-α similarly induced TLR2 gene expression in murine macrophages (101, 102), and acts is a key player in inflammation and atherosclerosis progression (103). Besides the role of cellular miRNAs in TLR signaling, authors recently reported that multiple viral miRNAs can activate production of proinflammatory mediators. For instance, Kaposi sarcoma herpes virus miRNAs such as miR-K-10b and miR-K12-12^*^ are involved in sepsis as agonists of TLR8 through secretion of IL-6 and IL-10 (104, 105). Furthermore, Epstein-Barr virus miRNAs such as BHRF1-1 are expressed at higher levels in patients with chronic lymphocytic leukemia than in healthy individuals, and these viral miRNAs can serve as prognostic biomarkers for cancer (106, 107). Overall, these studies highlight miRNAs as central drivers of the TLR expression through transcriptional regulation of them, as indicated in Table 1.

## How TLR Signaling may Modulate miRNA Expression

Initiation of the signaling cascade of immune response induced by TLR signaling can drive transcription of miRNAs during infection and inflammation. This is demonstrated by the fact that aberrant activation of TLR signaling after infection with microbial pathogens leads to dysregulation of miRNAs. Researchers have shown that infection of human peripheral blood monocytes (PBMCs) with *Francisella tularensis*, which is a highly pathogenic gram-negative bacterium that infects macrophages, induces expression of miR-155 in a TLR-dependent manner through downregulation of Src homology 2 domain-containing inositol 5-phosphatase 1 (SHIP1) (84). In concordance with this, authors reported significant differential expression of several miRNAs, including miR-155, after *F. tularensis* infection in primary human monocyte-derived macrophages and that *F. tularensis* infection leads to downmodulation of MyD88 and SHIP1 through an miR-155–dependent mechanism (64). Moreover, Leishmania RNA virus 1 was recognized by TLR3, and *Leishmania* infection induced miR-155 expression in murine bone-marrow macrophages (59). Concurrently, in that study, the pathogenesis of *LRV1*+ *Leishmania* infection decreased drastically in miR-155–deficient mice. In another study, let-7 and miR-98 were downregulated in murine macrophages upon Salmonella infection, whereas miR-155, miR-146a, and miR-21 were upregulated (77).

The immune-regulatory cytokine IL-10 may regulate transcription of miR-155 from the *BIC* gene in a signal transducer and activator of transcription 3-dependent manner in immortalized bone marrow-derived macrophages, and downmodulation of miR-155 expression leads to increased expression of SHIP1, which is one of the targets of miR-155 (71). Furthermore, investigators found that Ets2 is a critical transcription factor for the induction of miR-155 expression by LPS, and downmodulation of Ets2 leads to suppression of miR-155 by IL-10 (81). Another example miRNA signature is involved in human plasmacytoid DC activation (70), and miR-155 and its star form, miR-155^*^, were the most upregulated miRNAs in in primary human plasmacytoid DCs after TLR7 stimulation. MiR-155^*^ induced IFN-α/β expression by suppressing IRAK-M expression, whereas miR-155 suppressed TAB2 expression (70). TLR3-dependent antiviral as well as inflammatory activity can be regulated by IL-10, transforming growth factor-β, and miR-155 in non-parenchymal liver cells *in vitro* (67). Other studies of macrophages demonstrated that chronic alcohol exposure induces TNF-α secretion through increased miR-155 expression both *in vitro* and *in vivo* (108), and miR-155 deficiency can protect against alcohol-induced liver injury, oxidative stress, steatosis, and inflammation in miR-155–knockout mice (79). In a similar study, after chronic ethanol feeding, miR-155 induced TNF-α and MCP1 expression in the cerebellum in a TLR4-dependent manner (68). Researchers have also observed aberrant expression of miR-155 in macrophages after stimulation by poly(I:C) and IFN-β and that miR-155 expression is induced by other TLR ligands through MyD88- or TRIF-dependent signaling pathways (109). However, investigators found TLR-independent upregulation of mature miR-155 in the murine macrophage cell line J774A and murine primary bone marrow-derived macrophages during *Helicobacter pylori* infection (110). Authors reported that treatment with progesterone augmented LPS- and poly(I:C)-induced miR-155 expression in macrophages through inhibition of NF-κB activation and led to downmodulation of IL-6 and IFN-β production in TLR-activated macrophages by increasing SOCS1 expression (85). A recent study identified that miR-155 expression increased in monocyte-derived macrophages upon TLR3/4 but not TLR7 stimulation and that inhibition of miR-155 expression partially restored infectivity in poly(I:C)-treated monocyte-derived macrophages (69). Furthermore, miR-155 in PBMCs of systemic lupus erythematosus patients was specifically upregulated by high-mobility group box 1 protein in a MyD88-dependent manner during induction of anti-double-stranded DNA antibody, which is the central pathogenic autoantibody involved in pathogenesis of systemic lupus erythematosus (82). Authors reported that cold exposure (32°C) induced miR-155 expression in human monocytes and that increased miR-155 expression was associated with suppressed SOCS1 and SHIP1expression (65). Additionally, miR-155 was upregulated in ischemic cerebral tissue and promoted TNF-α and IL-1β expression by upregulating TLR4 and downregulating SOCS1 and MyD88 (63). Negative regulator proteins for the TLR4 pathway (IRAK-M, SHIP1, and SOCS1) were upregulated in Kupffer cells isolated from miR-155–deficient mice (58). Researchers have shown that miR-155-3p and miR-155-5p (the two mature miRNAs processed from the precursor miR-155 transcript) were highly expressed in mice after treatment with LPS, whereas expression of both was decreased in the lungs of triggering receptor expressed on myeloid cells 1- (TREM-1) knockout mice. Deficiency of TREM-1 significantly inhibited neutrophils and proinflammatory chemokines and cytokines, particularly IL-1β, TNF-α, and IL-6 (80). Researchers also identified that protein kinase Akt1 activated by LPS positively regulates the expression of let-7e and miR-181c but negatively regulates that of miR-155 and miR-125b revealing that let-7e inhibits the expression of TLR4, whereas miR-155 inhibits the expression of SOCS1; both proteins TLR4 and SOCS1 are critical for TLR signaling after LPS stimulation (95). In another study, investigators showed that exposure to angiopoietin-1 significantly decreased IRAK1 and TRAF6 protein expression but did not affect TLR4, MYD88, IRAK4, or TAK1 expression in human umbilical vein endothelial cells (88).

## The Ability of miRNAs to Bind to TLRs

MiRNAs may bind to TLRs and activate TLRs involved in intercellular communication in the tumor microenvironment (12, 13, 111). Authors reported that guanosine- and uridine-rich single-stranded RNA oligonucleotides derived from HIV-1 and influenza virus are recognized by murine TLR7 and human TLR8. Subsequently, activation of DCs and macrophages lead to the production of proinflammatory mediators such as IFN-α and cytokines (112, 113). Recently reported evidence demonstrated that extracellular vesicles such as exosomes and shed microvesicles isolated from different cell types may be novel mediators of cell-cell communication these vesicles can contain mRNAs, miRNAs, long non-coding RNAs, lipids, and DNA fragments. These active cargo molecules are packaged and released in exosome-derived cells and taken up by neighbor cells, where they are functionally active (114–116). MiRNAs are ubiquitously expressed in exosomes and are involved in modulation of the host immune response, expression of some activated molecules, enhanced tumor cell invasion, and mediation of intercellular communication (12, 117).

In 2012, researchers discovered that tumor-secreted exosomes in supernatants of lung cancer cells and exosomes loaded with miRNAs are physiological ligands for TLR7 and TLR8 (9, 12, 13). Expression of miR-21, miR-27b, and miR-29a was higher in exosomes derived from lung cancer cells than in those derived from HEK-293 cells (13). Upon co-culture of HEK-293 and RAW macrophages *in vitro*, labeled exosomes released from HEK-293 cells were incorporated with RAW macrophages, and miR-29a co-localized with TLR7 and TLR8 in the RAW macrophages (9, 12, 13). In addition, these investigators reported that cancer cell-derived exosomal miRNAs can bind to and activate TLR8 in macrophages and stimulate TLR8-mediated activation of NF-κB and NF-κB–mediated release of the proinflammatory and prometastatic cytokines IL-6 and TNF-α (13). Therefore, malignant cells release signals via exosomes loaded with miRNAs to the surrounding cells in their microenvironments that promote tumorigenesis and dissemination by different TLRs in humans (TLR8) and mice (TLR7) (13, 118). In our recent study, we demonstrated that exosomal miR-1246 released in abundance from ovarian cancer cells and miR-1246 transmit molecular signals to M2-type macrophages but not M0-type macrophages by shuttling exosomes (119).

Moreover, treatment with liposome-encapsulated miR-21 significantly induced human TLR8 expression in hematopoietic cell lines and PBMCs obtained from patients with systemic lupus erythematosus (90). Single-stranded RNAs containing twenty-nucleotide guanosine- and uridine-rich regions derived from human immunodeficiency virus and the influenza virus are physiological ligands for TLR7 and TLR8 that bind to TLR and activate TLR signaling (113, 120). Furthermore, the let-7 family contains a specific GU-rich motif GUUGUGU, which is present in the core of single-stranded RNA40 and responsible for murine TLR7 activation (15, 113, 120). Researchers recently discovered that let-7 may interact with TLR7 and activate TLR signaling in murine macrophages and microglia (15). They found that six nucleotide exchanges in the seed sequence of let-7b dramatically diminished induction of TNF-α expression in microglia and macrophages. In addition to let-7b, let-7a, −7c, and −7g induced a dose- and time-dependent cytokine response in wild-type immortalized bone marrow-derived macrophages but not TLR7-deficient cells (15). Consistent with these findings in macrophages, these investigators showed that neuronal loss was induced by let-7a, let-7c, let-7g, and miR-599 through TLR7. To further understand the role of let-7 in neurodegeneration *in vivo*, Lehmann et al. (15) showed that treatment with let-7b significantly induced marked axonal injury and neuronal loss in wild-type mice, whereas mutant let-7b rescued this phenotype. In contrast, TLR7 deficiency in Tlr7 knockout mice can protect against let-7b's induced neurotoxic effects. These findings suggest that endogenous miRNAs such as let-7b can be released during neuroinflammation and may cause further spread of central nervous system damage in patients with neurodegenerative disorders such as Alzheimer disease (15, 121).

## Conclusion and Future Perspectives

MiRNAs are strongly implicated to have roles in the development and progression of inflammation-related diseases. Increasing numbers of studies have identified that miRNAs can act as physiological ligands for TLRs. The next challenges are to understand the complex mechanisms behind these integrated networks of interactions and, more importantly, determine whether therapeutic modulation of TLR-regulated and TLR-regulating miRNAs is beneficial for patients with cancer or inflammatory diseases. At present, MRG-106 therapy, is an oligonucleotide inhibitor of miR-155, for being tested in phase 1 clinical studies in Cutaneous T-cell Lymphoma, Mycosis Fungoides, Chronic Lymphocytic Leukemia, Diffuse Large B-Cell Lymphoma and Adult T-Cell Leukemia/Lymphoma (clinicaltrials.gov identifier NCT02580552). Therefore, researchers expect more preclinical and clinical studies regarding this new therapeutic avenue (93, 122, 123).

## Author Contributions

RB, MB, and GC wrote the first draft of the manuscript and contributed to the writing of the manuscript.

### Conflict of Interest Statement

The authors declare that the research was conducted in the absence of any commercial or financial relationships that could be construed as a potential conflict of interest.

## Abbreviations

AGO2: argonaute RISC catalytic component 2
CIS: cytokine-inducible Src homology 2-containing protein
DCs: dendritic cells
HMGB1: high-mobility group box 1
HSV-1: herpes simplex virus type 1
IFNs: interferons
IL: interleukin
IRAK1: interleukin 1 receptor associated kinase 1
IRF: interferon-regulatory factor
KSRP: KH-type splicing regulatory protein
LPS: lipopolysaccharide
MCP1: monocyte chemoattractant protein-1
miRNA: microRNA
mRNA: messenger RNA
MYD88: myeloid differentiation primary response protein
NF-κB: nuclear factor κB
PBMC: peripheral blood mononuclear cells
PDCD4: programmed cell death 4
PDCs: plasmacytoid dendritic cells
ROS: reactive oxygen species
SHIP1: inositol polyphosphate-5-phosphatase D
SNAP23: synaptosome associated protein 23
SOCS1: suppressor of cytokine signaling 1
TAB2: TGF-beta activated kinase 1 binding protein 2
TGF: transforming growth factor
TICAM: TIR domain-containing adapter molecule
TIR: Toll/interleukin-1 receptor
TLRs: Toll-like receptors
TNF: tumor necrosis factor.
